# Does hysteroscopic metroplasty for septate uterus represent a risk factor for adverse outcome during pregnancy and labor?

**DOI:** 10.1007/s10397-015-0916-2

**Published:** 2015-10-31

**Authors:** Nataša Kenda Šuster, Marco Gergolet

**Affiliations:** Department of Obsterics and Gynecology, University of Ljubljana, Šlajmerjeva 3, 1000 Ljubljana, Slovenia; Casa di Cura Sanatorio Triestino, Via Rossetti 62, 34141 Trieste, Italy

**Keywords:** Hysteroscopic metroplasty, Septate uterus, Adverse obstetric outcome, Metroplasty complications

## Abstract

The aim of the study was to evaluate whether hysteroscopic metroplasty for septate uterus represents a risk factor of adverse outcome in pregnancy, during labor, and after delivery. This is a retrospective comparative study of obstetric complications of 99 patients who underwent hysteroscopic metroplasty in a 5-year period (study group) and 4155 women, who gave birth in the same hospital in the same period (control group). No difference in obstetric outcome (preterm labor, hemorrhage before and after delivery, mean weeks of gestation at delivery, mean birth weight, breech presentation, and cesarean section rate) between the two groups has been found. The results of this study suggest that patients who underwent hysteroscopic metroplasty for septate uterus are at no higher risk of adverse obstetric outcome at term and during labor, comparing to the general population. Though vaginal delivery seems to be safe, rare but serious complication, reported by several studies, like uterine rupture during pregnancy or labor, should always be taken into consideration.

## Introduction

Septate uterus is the commonest congenital uterine anomaly and is a known factor of infertility as well as cause of first and second trimester spontaneous miscarriage and preterm delivery [1–4]. Fetal malpresentation and placentar anomalies have also been reported [5, 6].

Hysteroscopic metroplasty for septate uterus seems to be a simple and relatively safe procedure, but several complications can occur either during the procedure or in subsequent pregnancy and childbirth. In a series of 600 metroplasties, Shveiky reported a 3 % rate of complications, including intra-operative perforations of uterus which occurred in 1 %. Two thirds of complications were related to the cervical dilatation or the insertion of the resectoscope [7]. Agostini analyzed the incidence of early complications in 2116 consecutive operative hysteroscopies. In his series, the rate of perforation resulted to be 1.6 %. After stratifying the data related to the uterine pathology, lysis of synechiae was the procedure with the highest relative risk for perforations, whereas other procedures, such as septa resection, registered a lower incidence of complications. Endometrial ablation, polyp resection, and myoma resection did not cause significant rate of complications whatsoever. Postoperative hemorrhage has been reported in less than 1 % of cases and was mostly self-limiting. The hemorrhage occurred more frequently in case of synechiolisys than in other procedures such as myoma or polyp ablation and septum resection [8]. Complications related to the procedure can manifest during successive pregnancy, labor, and delivery. According to Agostini, patients who underwent hysteroscopic metroplasty are at increased risk for fetal malpresentation at term, low birth weight infants, and delivery by cesarean section [9]. The dilatation of the cervix may disrupt fibers in the cervix which can lead to the cervical-isthmical insufficiency [10]. The incision at the fundus over a security range of 10 mm could cause weakening of the myometrial layer at the fundus, with a subsequent risk for rupture during pregnancy and labor [11]. Perforation of uterus during the procedure and use of monopolar current could represent a predisposing factor for rupture of the uterus during labor [12–14]. On the other hand, cases of uterine rupture after metroplasty have been described independently from used method for septum resection (bipolar or monopolar energy, laser, cold scissors) [12, 14, 15].

The aim of the present study was to verify if hysteroscopic metroplasty for septate uterus represents a risk factor for any minor or severe complication during the subsequent pregnancy and labor.

## Materials and methods

Study group consisted of 99 patients who underwent hysteroscopic metroplasty in a 5-year period, between January 2006 to December 2011 at the General Hospital »dr. Franc Derganc« in Nova Gorica, Slovenia, and delivered in the same facility. Labor outcomes of the study group were compared to the labor outcomes of 4155 women (control group) who gave birth in the same period and in the same hospital. Only singleton pregnancies and first delivery after metroplasty were considered. An 8-mm monopolar Karl Storz resectoscope with 1.5 % glycine solution as a distension medium was used. The authors (M.G. and N.K.Š.) performed all the metroplasties. After achieving good visibility, the ostia were taken as orientation points and the procedure was stopped when the fundus was aligned with the tubal ostia or when small arterial blood vessels became visible at the myometrial layer. In order to perform the metroplasty in the early proliferative phase, synchronization of the cycles was done by giving a contraceptive pill for at least 21 days. Five to 7 days after onset of the withdrawal bleeding, the procedure was performed. No intra-operative complication was recorded. All patients underwent vaginal ultrasound 1 month after surgery to exclude a residual septation. In case of an unclear ultrasound findings, “office” hysteroscopy was carried out. A database file was set up using Microsoft Excel for Windows (Redmond, WA, USA) to facilitate data of entry and retrieval.

### Statistical analysis

Data on deliveries and newborns have been obtained from the National Perinatal Informative System of Slovenia (PERIS), a national register including data on deliveries and newborns of all the 13 delivery wards in the Country. SPSS/PC 17.0 program was used for statistical analysis. Student’s *t* test was used for the analysis of quantitative variables; qualitative differences between the two groups were analyzed by the Pearson’s *χ*^2^ test.

All procedures followed were in accordance with the ethical standards of the responsible committee on human experimentation (institutional and national) and with the Helsinki Declaration of 1975, as revised in 2000.

The study was observational and retrospective, so an informed consent was not necessary.

## Results

In 60 patients (60.6 %), the indication for metroplasty was unexplained primary infertility or prolonged secondary infertility. In 32 (32.3 %) patients, the procedure was performed in course of artificial reproduction technique (ART) workout, which according to data from literature increases the chances of ART success [16, 17]. Data on obstetric outcome are represented in Table 1. Two hundred six (206) patients in the control group have had a previous CS (5.0 %), whereas we recorded two CS out of nine deliveries before metroplasty in the study group. One woman in the study group (1 %) and 17 women in the control group (0.4 %) delivered before 32nd week of gestation (n.s.). Mean week of gestation at delivery was similar in both groups (39.21 ± 2,4 vs. 39.47 ± 1.6 weeks). In the study group, 19 CS were recorded (18.2 %) vs 659 CS in the control group (15.9 %) (n.s.). In the study group, 12 cases of CS were done because of dystocia, 5 cases due to an acute fetal distress, and in 3 cases, the indication was malpresentation (breech presentation), whereas in 2 cases, the indication for CS was a pathological condition of the mother. The rate of breech presentation was similar in both groups (3 vs. 3.9 %). Uterine ruptures were not recorded in the study group, whereas in two cases in the control group, a partial rupture in the previous cesarean section scar were reported during second cesarean section. In both cases, the ruptures were asymptomatic and diagnosed incidentally during the procedure.

**Table 1:** Outcome of pregnancy

Variable	Study group (*n* = 99)	Control group (*n* = 4155)	*p*
Preterm delivery total (<37 WG)	8 (8.1 %)	178 (4.4 %)	0.085
Preterm delivery (≤32 WG)	1 (1 %)	17 (0.4 %)	0.366
Mean week of gestation ± SD	39.21 ± 2.4	39.47 ± 1.6	0.122
Cesarean section	19 (19.2 %)	59 (15.9 %)	0.371
Mean birth weight (g) ± SD	3405 ± 430	3453 ± 466	0.330
Breech presentation	3 (3 %)	161 (3.9 %)	0.666
Placental abruption	1 (1 %)	40 (1 %)	0.962
Placenta praevia	0 (0 %)	3 (0.1 %)	0.782
Early postpartum hemorrhage	2 (2 %)	26 (0.6)	0.090
Uterine atony	2 (2 %)	73 (1.8 %)	0.844
Retained placental fragments	2 (2 %)	41 (1 %)	0.310
Adherent placenta	2 (2 %)	39 (0.9 %)	0.276
Late postpartum hemorrhage	0 (0 %)	5 (0.1 %)	0.730
Uterine rupture	0 (0 %)	2 (0.04)	0.833

*WG* weeks of gestation

The incidences of placental abruption (1 vs. 1 %), early postpartum hemorrhage (2 vs. 0.6 %), uterine atony (2 vs. 1.8 %), retained placental fragments (2 vs. 1 %), adherent placenta (2 vs. 0.9 %), and late postpartum hemorrhage (0 vs. 0.1 %) did not reach the statistical significance.

## Discussion

According to numerous papers, septate uterus could be the cause of repeated miscarriage, second trimester pregnancy loss, and fetal malpresentations. Septate uterus is not generally thought to be a cause of infertility, although several papers report a longer time to conceive in this case. Similarly, uterine septum may impair the results of assisted reproductive techniques [18–21].

In a systematic review and meta-analysis of comparative studies, Venetis reported an increased relative risk (RR) for miscarriage in women with congenital uterine anomalies (CUA) compared to women with normal uterus. A higher RR for preterm delivery (2.21), malpresentation at delivery (RR 4.75), low birth weight (RR 1.93), and perinatal mortality rates (RR 2.43) were reported to be significantly higher in women with CUA.

After metroplasty, the probability for spontaneous miscarriage was reduced (RR 0.37) compared with untreated women [22].

A slight but not significant increase of cesarean section rate has been found in the study group. As we already mentioned, this group consisted mainly of infertile women, whose pregnancies are considered frequently at high risk, since obtained after years of infertility or several miscarriages. Subsequently, a more cautious approach to delivery could be a possible explanation for higher incidence of operative delivery. Previous uterine surgery, uterine septa resection, could also be another indication for CS.

Metroplasties were performed on patients who suffered from previous pregnancy loss or infertility. Several studies reported an increased incidence of premature labor in patients who underwent dilatation and curettage or conceived after a long time of infertility [23, 24]. Though no statistically significant differences have been found between the groups in our study, there was a slight increase of the premature delivery rate in the study group (7.1 %). To compare, the preterm delivery rate in general population varies between 12 to 13 % in the USA and 5 up to 9 % in other developed countries [25].

Agostini reported an incidence of 35 % of breech presentation in patients with a 10-mm residual septum after metroplasty [9]. No differences in the rate of breech presentation between the groups have been found in the present study. Placenta accreta has been reported to be a possible complication of uterine surgery such as hysteroscopic myomectomy and lysis of intra uterine synechiae [26]. No study in the literature reports any case of placenta accreta or increta after metroplasty, and no case has been observed in the present study. According to Sentilhes, uterine surgery using monopolar energy could be a possible factor of weakening of the uterine wall, which could consequently cause uterine rupture during labor. The author concludes that the observation of a security limit of the thickness of the myometrium in case of using monopolar current cannot be enough. In order to avoid uterine rupture, he suggests the use of bipolar energy [27]. According to several studies, uterine perforation during surgery seems to be the most important prognostic risk factor for uterine rupture in pregnancy and during labor [28]. In an extensive review, Valle and Ekpo report different complications in course of surgery. Uterine perforations occurred either in case of using resectoscope or small diameter hysteroscope [29]. Argumentations about higher incidence of surgical complications in case of using resectoscope instead of small diameter scopes with micro scissors or Versapoint seem to be more subjective impressions of some authors rather than evidence-based [10, 30].

Randomized controlled trials are needed to confirm the beneficial effects of metroplasty reported by numerous non-randomized papers [31]. Such trials are in our opinion difficult to carry on, not only because of a relative difficulty to randomize patients but also because of an objective difficulty to design an appropriate study [32].

In the era of internet, women with septate uterus who are aware of dozens of non-randomized publications which confirm the improvement of pregnancy and live birth rates after metroplasty would decline to enter the randomization. Moreover, a multicentre randomized trial would be difficult because of a lack of uniformity in the discrimination between a normal uterine cavity and a small septate uterus. The new ESHRE-ESGE classification on congenital uterine anomalies may result as a helpful tool for reaching more homogeneity in studies especially in the interpretation of uterine cavity imaging, since the terminology “arcuate” uterus has been excluded by this classification [33].

## Conclusions

Hysteroscopic metroplasty seems to significantly improve the obstetric outcome in a population of women with previous miscarriage. The procedure seems to be safe when done by skilled surgeons [34, 35].

While waiting for the highest level of evidence, according to prospective and retrospective epidemiological studies, it seems that hysteroscopic resection of uterine septum may improve either the pregnancy outcome or the outcome of ART techniques and may shorten the time to conceive in couples with primary or secondary infertility [18–21, 31, 36].

In experienced hands, the procedure seems not to be harmful for patients and not to threaten the future pregnancy and labor.
